# Programmable Biosensors Based on RNA-Guided CRISPR/Cas Endonuclease

**DOI:** 10.1186/s12575-021-00163-7

**Published:** 2022-01-23

**Authors:** Xiaolong Liu, Mubashir Hussain, Jianguo Dai, Yonghong Li, Lijun Zhang, Jian Yang, Zeeshan Ali, Nongyue He, Yongjun Tang

**Affiliations:** 1 Shenzhen Key Laboratory of Fermentation, Purification and Analysis, Shenzhen Polytechnic, Shenzhen, 518055 China; 2 State Key Laboratory of Bioelectronics, School of Biological Science and Medical Engineering, Southeast University, Nanjing, 210096 China

**Keywords:** CRISPR/Cas, Nucleic acids, Pathogen, Diagnostic, Biosensors

## Abstract

Highly infectious illnesses caused by pathogens constitute severe threats to public health and lead to global economic loss. The use of robust and programmable clustered regularly interspaced short palindromic repeat and CRISPR-associated protein (CRISPR-Cas) systems, repurposed from genome-engineering applications has markedly improved traditional nucleic acid detection for precise identification, independently enabling rapid diagnostics of multiplex biomarker with genetic and mutation related to tumors, and microbial pathogens. In this review, we delineate the utility of the current CRISPR-Cas enzyme as biosensors by which these effector toolkits achieve recognition, signaling amplification, and finally, accurate detection. Additionally, we discuss the details of the dominance and hurdles related to expanding this revolutionary technology into an effective and convenient contraption crucial for improving the rational redesign to CRISPR/Cas biosensing. Overall, this review provides an insight into the current status of rapid and POC diagnostic systems by CRISPR/Cas tools.

## Introduction

In general, infectious illness outbreaks caused by pathogens exhibit several common and prominent features such as high infectivity, widespread distribution, and an incubation period, thereby seriously threatening public health and leading to global economic loss. Notable examples throughout human history including pathogenic pulmonary tuberculosis, Spanish flu, H1N1 flu, Middle East respiratory syndrome (MERS) [1–3], severe acute respiratory syndrome (SARS) [4], and more recently SARS-CoV-2 [5, 6] have led to catastrophic effects for individuals and society accompanied with disability, casualties, factory closures, and economic stagnation [7]. Thus, rapid and accurate identification of infectious and non-infectious diseases is essential to optimize clinical care and guide infection control and public health interventions to limit spread of illness both in highly specialized medical centers and remote health care settings. For pathogens, the ideal diagnostic test would be inexpensive, accurate, rapid, and allow for point-of-care use on multiple specimen types without the need for technical expertise or complicated ancillary equipment. Over the past decades, epidemic prevention has predominantly dependent on molecular diagnostics, typically involving enzyme-linked immunosorbent assays (ELISA) based on antigen-antibody tests or nucleic acid detection (e.g., real-time or reverse transcription (RT) polymerase chain reaction (PCR)). However, common complications in such tests include not only the requirement for complicated genotyping but also for intricate devices and intricate, fastidious processes, which represents a harsh burden for undeveloped areas and those with poor hygienic conditions. As an alternative, recent advances in clustered regularly interspaced short palindromic repeat and CRISPR-associated protein (CRISPR-Cas) technologies offer considerable promise for precisely and indirectly detecting infectious pathogens, along with extended applications in tumor diagnostics via preamplification using recombinase polymerase amplification (RPA) combined with Cas nuclease functionality for recognition of single bases together with collateral *trans*-nuclease activity [8, 9]. Moreover, multifarious approaches utilizing programmable Cas detection systems have been developed as highly sensitive and rapid tools in various fields for in vivo diagnostics. For example, direct applications based on Cas protein without preamplification include detection methods incorporating Cas9 protein [10–12]. In contrast, colorimetric DNA detection platforms and fully microfluidic Ebola virus detection were performed using Cas12 and Cas13, respectively [13, 14].

Among the established CRISPR/Cas-based nucleic acid biosensing systems, the fundamental differences relate to implementing different Cas effectors (Cas9, Cas13, Cas12, and Cas14), although the combination of various components is also observed. Specifically, the currently reported CRISPR/Cas detecting systems can be classified into three types according to the different Cas proteins. Broadly, Cas effectors are divided into two classes based on structural features and mechanisms of CRISPR–Cas. For class 1 systems, multiple Cas proteins bind crRNA to form a surveillance complex. In contrast, in class 2 systems, mainly developed as CRISPR/Cas biosensing platforms, a single multidomain Cas protein binds a guide RNA responsible for CRISPR RNA (crRNA) interference [15]. Class 1 systems include Types I, III, and IV; class 2 systems contain Types II, V, and VI. Molecular diagnostic applications with excellent potential and new developments include Cas9, which allows RNA-guided adaptive immune systems to protect archaebacteria against invading exogenous genomic DNA. The technique has been widely applied to cell and model establishment, functional genome screening, gene regulation, chromosome active marker imaging, and gene therapy. Similar to Type II systems (Cas9 and its mutants), Types V-A, B (Cas12, formerly called Cpf1), both with target activity driven by dsDNA, and Type VI-A, B, D (Cas13, also named C2c2 driven by ssRNA) along with all associated orthologs were reported to independently exhibit potential as biosensors to provide precise biomarker detection based on their ability to process sequence-specific target identification and trigger collateral *trans*-DNA/RNA cleavage [16–21]. Typical establishments and comparisons of major properties based on the different Cas effectors for these three classes are listed in Table 1. In particular, various CRISPR-Cas strategies incorporating Cas9 have been rapidly developed for use in pathogen diagnosis. Extensive research has also been conducted to discover novel Cas variants and unveil their functions to open up additional novel applications.

**Table 1:** Comparison of Classified platforms incorporating CRISPR/Cas effectors

Classifications	Effector	System	Recognition type	Sensitivity	Specificity	Signal amplification	Quantitative	Multiplex	Time	Readout	Infrastructure requirement	Reference
Cas9-based class	Sp-dCas9	RCH	dsDNA	fM	1 nt	RCA	Y^a^	N^a^	< 4 h	TMB	substantial	[22]
		PC reporter	dsDNA	One copy	NA	PCR	N	N	10 mins*	Luciferase	Few	[23]
Cas13-based class	CcaCas13b PsmCas13b LwaCas13a	SHERLOCKv2f	dsDNA/Virus RNA	aM	1 nt	RPA	Y	Y	2 ~ 5 h	Fluorescence	Substantial	[15]
Cas12-based class	LbCas12a	DETECTR	HPV	aM	1 nt	RPA	N	N	~ 1 h	fluorescence	substantial	[24]
	LbCas12a	Cas12aVDet	Mycoplasma	aM	1 nt	RPA	N	N	~ 30 min	Naked eye	Few	[25]
	LbCas12a	DETECTR-COVID19	SARS-CoV-2	95%	1 nt	RT-LAMP	N	N	~ 45 min	LFA	Few	[19]
Cas14-based class	Cas14a1	Cas14-DETECTR	ssDNA	NA	1 nt	PTA^a^	N	N	NA	fluorescence	substantial	[26]

^a^All features were claimed by the original publications; Y: yes; N: no; PTA: phosphorothioate amplification; NA: uncertainty;

* shown 10 mins after PCR

### Development in the Sensor Capabilities of CRISPR/Cas9

Type II CRISPR systems utilize a single DNA endonuclease, Cas9 from *Streptococcus pyogenes* (SpCas9) to effect target recognition propelled by complementarity matching between spacer sequence from crRNA and target double-stranded (ds) DNA followed by cleavage of dsDNA substrates [27]. The process is driven through contact with two unrelated nuclease domains depicted as RuvC (ribonuclease (RNase) H-like fold) and HNH (histidine–asparagine–histidine), which are responsible for the active Cas9 conformation and selectively triggering nuclease domain activity, respectively [28]. Thus, CRISPR-Cas Cas9 possesses a programmable property potentially suitable for target dsDNA detection because of the ease of changing target specificity by altering the guide RNA sequence (gRNA). In particular, following the establishment of the Cas9 off-target mutagenesis landscape, considerable research interest was raised toward programming the CRISPR-Cas9 system to enhance its specificity while simultaneously ensuring accuracy for application to various diagnostic fields. One strategy was to rationally engineer *Sp*Cas9 to construct different variants by applying crystal structure to screen non-target DNA contacts and other non-specific binding. Two derivatives, high-fidelity (SpCas9-HF1) and enhanced specificity [eSpCas9] Cas9, have been shown to exhibit precise genome editing in human cells with reduced off-target mutations using both genome-wide and targeted sequencing methods [29, 30]. Further structural and biophysical analyses of these variants identified a noncatalytic domain, REC3, within Cas9 that could be utilized to allosterically regulate global Cas9 conformational changes [28]. Disruption of this domain led to a Cas9 variant (HypaCas9) that exhibits hyper-accurate performance without compromised efficiency in human cells [31]. Other structural derivatives of *sp*Cas9 include dCas9 (deactivated Cas9), alternatively termed d*Sp*Cas9 (involving the Cas9 D10A nickase Cas9n). The well-characterized *sp*Cas9 carrying a mutation of the HNH catalytic residue (*sp*Cas9-H840A nickase) generally created using a double nicking strategy, in addition to the dead Cas9 silencing mutations of D10A and H841A (dCas9), which retains intact programmable DNA-binding activity [32–34]. A detailed discussion of the various strategies based on Cas9 and its derivatives as mutant effectors, including unpractical methods, is provided in the following sections.

### Cas9-Based Double-Strand Cleavage

**Fig. 1:**
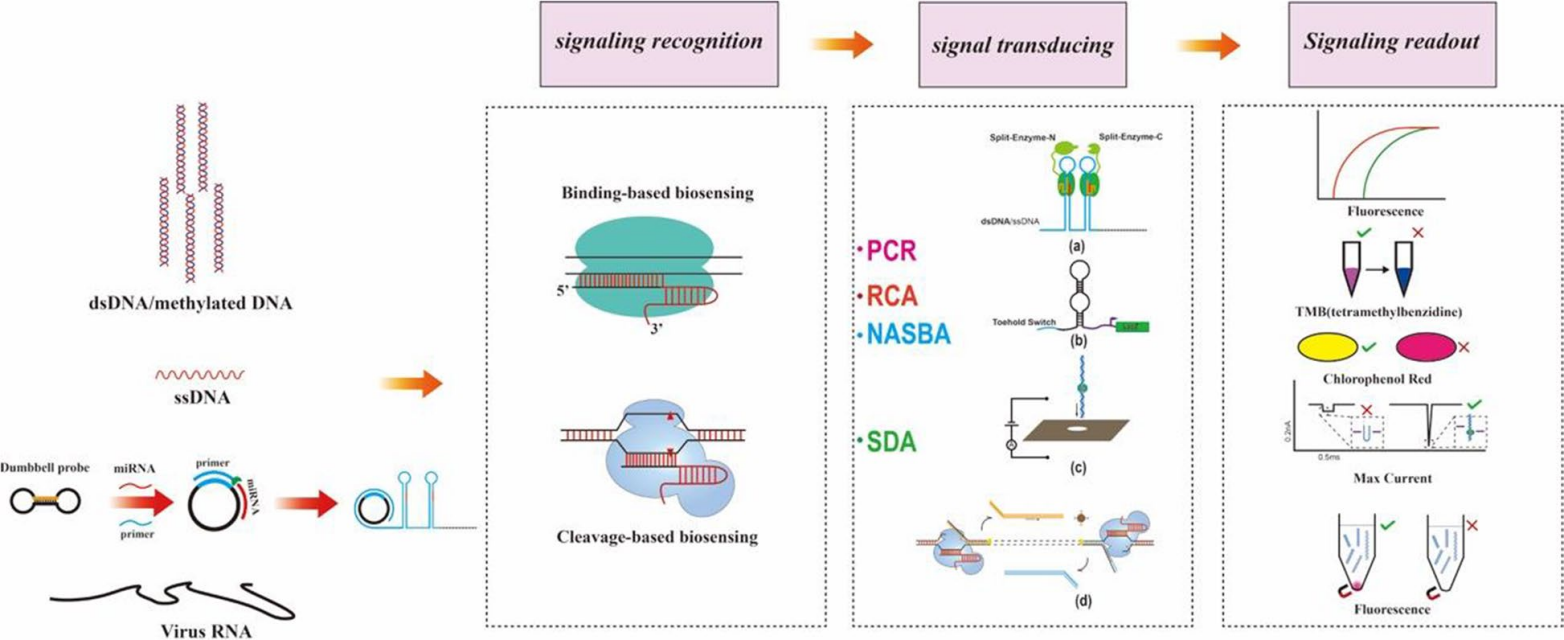
Schematics for Cas9-based Biosensing Systems

The improvement of traditional nucleic acid amplification to genotype can be achieved by combining SpCas9 and its derivative, precise in DNA cleavage in vitro. Despite the wide use of traditional strategies to detect pathogens in clinical diagnostics including food safety, medicolegal contexts, and genotyping; specific diagnoses depend on symmetrical or asymmetric primer-paired template DNA, the fidelity of conventional Taq DNA polymerase, and strand displacement polymerase, instead of accurate recognition of high contents of GC. Moreover, SNP sites still lead to non-specific amplification. However, template DNA could, prior to capture and enrichment for Cas9/sgRNA, act as “caretaker” to control nucleic acids amplification, owing to high cleavage activity hinged on distinguishing single base and sensitivity to PAM sequence. CUT-PCR (CRISPR-mediated, ultrasensitive detection of target DNA-PCR) method was successfully applied in cancer diagnosis at early stages, through inactive Cas9 without PAM sequence to discriminate wild-type DNA from mutant DNA, which recognized ctDNA (circulating tumor DNA) of colorectal cancer patient’s blood [35]. Besides, PCR-based applications using CRISPR/Cas9 reportedly has the potential to detect gene mutation efficiently even in low-frequency conditions of cfDNA detection, increasing the sensitivity of CRISPR/Cas9 cleavage-based PCR and blocker PCR. Moreover, named after CRISPR-typing PCR (ctPCR1.0), CARP (Cas9/sgRNAs-associated reverse PCR) ctPCR3.0 combined with Cas9 was developed to detect the L1 and E6-E7 genes of two high-risk HPVs, HPV16 and HPV18, in the genomic DNA of two HPV-positive cervical carcinoma cells (HeLa and SiHa) [36, 37]. These new CRISPR/Cas9-based PCR system applications provide a potential for heterogeneous specimens in clinical diagnosis and treatment management. Although the specificity of the ctPCR method is improved compared to that of conventional PCR and its derivative technology, this method is inferior in both operational efficiency and cost. To sensitively detect dsDNA in the CAS-exponential amplification reaction (EXPAR) system [38], the exposed blunt-ended cleavage site is essential for driving exponential amplification reaction, quantified by measuring the product-related fluorescence signal. Indeed, *Sp*Cas9 provides the cleavage function as a novel on-off tool. First, nicking triggered exponential amplification reaction (NTEXPAR) exhibits a versatile and highly efficient pre-screening of sgRNAs to analyze *Sp*Cas9 cleaved dsDNA in genome editing [39, 40]. Subsequently, improvement of NTEXPAR combined with Cas9, with cleavage started by PAMmer (the design of a single antisense PAM oligonucleotide) without the limits of PAM sequence, was successfully developed to detect DNA methylation and *L. monocytogenes* total RNA; this strategy obtained a detection limit of 0.82 amol and specificity in the recognition of single-base mismatch, as well as providing a new paradigm for molecular diagnostic. Furthermore, nucleic acid sequence-based amplification-CRISPR cleavage (NASBACC) system is portable, low-cost diagnostic platform, that genotypes American ZIKV, African ZIKV, and Dengue virus by using colorimetric detection to achieve low femtomolar detection in infected monkey plasma, critically through identified PAM site that guides the amplification of Toehold sequence via cleavage by CRISPR/Cas9 (Fig. 1b) [17].

### dCas9-Based Inactivation of Double-Strand Caught

In addition to harnessing the enzymatic cleavage activity of Cas9 effector, other structural derivatives of *sp*Cas9, namely dCas9, produced by mutants of *Sp*Cas9 involve double nicking and single nicking strategies, which retain intact programmable DNA-binding activity and accurate target recognition [33, 34]. Thus, steady CRISPR/Cas9-based binding to accurate target DNA can be a key tool for signal recognition in detecting nucleic acids. In particular, the double nicking strategy provides more creative key applications. The enzymatically deactivated Cas9 effector (dCas9) with sequence-specific binding capability has also been employed to develop several new CRISPR/Cas-based nucleic acid biosensing systems that are similar to conventional in situ hybridization methods using transcription activator-like effectors (TALE) or zinc finger protein [41, 42]. Especially, paired dCas9 (**PC**) reporter system detected *Mycobacterium tuberculosis* DNA using a pair of dCas9 enzymes linked to split halves of luciferase [23]. Bioluminescence signal produced by colocalization of split fragments leads to completely active luciferase when the reporter is paired with a ~  44 bp target sequence defined by sgRNAs (Fig. 1a). Similarly, another example is based on rolling circle amplification (RCA)-CRISPR-split-HRP **(RCH)** system for miRNA detection. Specifically, target colocalization of two dCas9 protein linked to a split HRP (horseradish peroxidase protein), guided by an on-off HRP enzyme, can generate a colorimetric signal readout by the addition of the chromogenic substrate tetramethylbenzidine (TMB), and if the target DNA is present, the signal could be amplified by RCA [22] (Fig. 1a). Moreover, the nanopore/dCas9 system was developed to detect target DNA using solid-state nanopore. According to the report, even at a high salt condition (1 M LiCl), dCas9 proteins were found to remain stably bound [43]. Although the sensitivity of the nanopore/dCas9 system remains indistinct, there is support for its advanced details in various applications in the future; the nanopore-based CRISPR/dCas9 biosensing approach can be potentially integrated with a portable device in DNA-typing based diagnostics (Fig. 1c). Recently, as a single nicking strategy, Zhou and colleagues reported CRISPR–Cas9-triggered nicking endonuclease-mediated isothermal strand displacement amplification (CRISDA) system, similar to conventional SDA strategy [44]. The CRISDA system utilizes the single-strand cleavage property of Cas9(H840A)/sgRNA on the target DNA and replaces conventional SDA’s thermal denaturation treatment, initiating isothermal amplification. Eventually, Cy5-labeled and biotin-labeled PNA (peptide nucleic acid) probes are specifically applied as reporter and capture enrichment, respectively, for signal readout (Fig. 1d).

## Features and Applications of Cas13 Effectors

### Dual RNase Activities of Cas13

Unique single-subunit RNA-guided Cas 13 proteins studied to date possess dual distinct RNase activities chemically and mechanistically different from CRISPR enzyme Cpf1, Cas9 [24, 28, 45–47]. It is a simpler guide-RNA that does not need trans-activating crRNA. Additionally, binding to a complementary single-stranded RNA (ssRNA) sequence adjacent to a protospacer flanking sequence (PFS, analogous to PAM) activates the higher eukaryotes and prokaryotes nucleotide-binding RNase (HEPN) domain nuclease to indiscriminately cleave the bound RNA target (*cis*-cleavage) and other ssRNA in solution (*trans*-cleavage) [28, 45]. Thus, there are critical hints potentially allow sensitive detection based on ssRNA biomarker, such as virus, tumor miRNA [15, 20]. Basically, the activity of HEPN-nuclease is inhibited by partial occlusion of the HEPN active site until binding to the activator-RNA occurs, effectively rendering the activator-RNA the allosteric switch for RNase activity that is critical point of specificity in methods itself for accurate recognition of targeting ssRNA [28]. Otherwise, Cas13a of spacer recognizing target sequences of 22 ~ 28 nt complementary at crRNA activates ssRNA cleavage that must contain one at least 24 nt-long stem-loop structure and had bias to A, U, or C, but inhibited RNase by 5′-G at PFS [48–50]. Additionally, Cas13b system, Cas13a homologue, might be more specific than Cas13a because RNA targeting is dependent on a double-sided PFS with a D (A, U, or G) at 5′ end and NAN/NNA at 3′end [51]. Especially, in this system, a single mismatch across the spacer can be tolerated, but two mismatches distributed in the central region of the spacer can dramatically reduce the target RNA cleavage efficiency [15, 52–54]. At this point, low intrinsic specificity of recognition formatted to single strand crRNA is not enough to complete than traditional PCR by two-paired primer in triggered strategy of nucleic amplification. But designed process of guide-RNA performs by currently software and website are simpler. Thus, these recent advances have uncovered a mechanism for non-specific RNase activity by RNA target recognition, which has since been used for RNA detection applications.

### Application of Cas13 Proteins for Nucleic Acid Detection

**Fig. 2:**
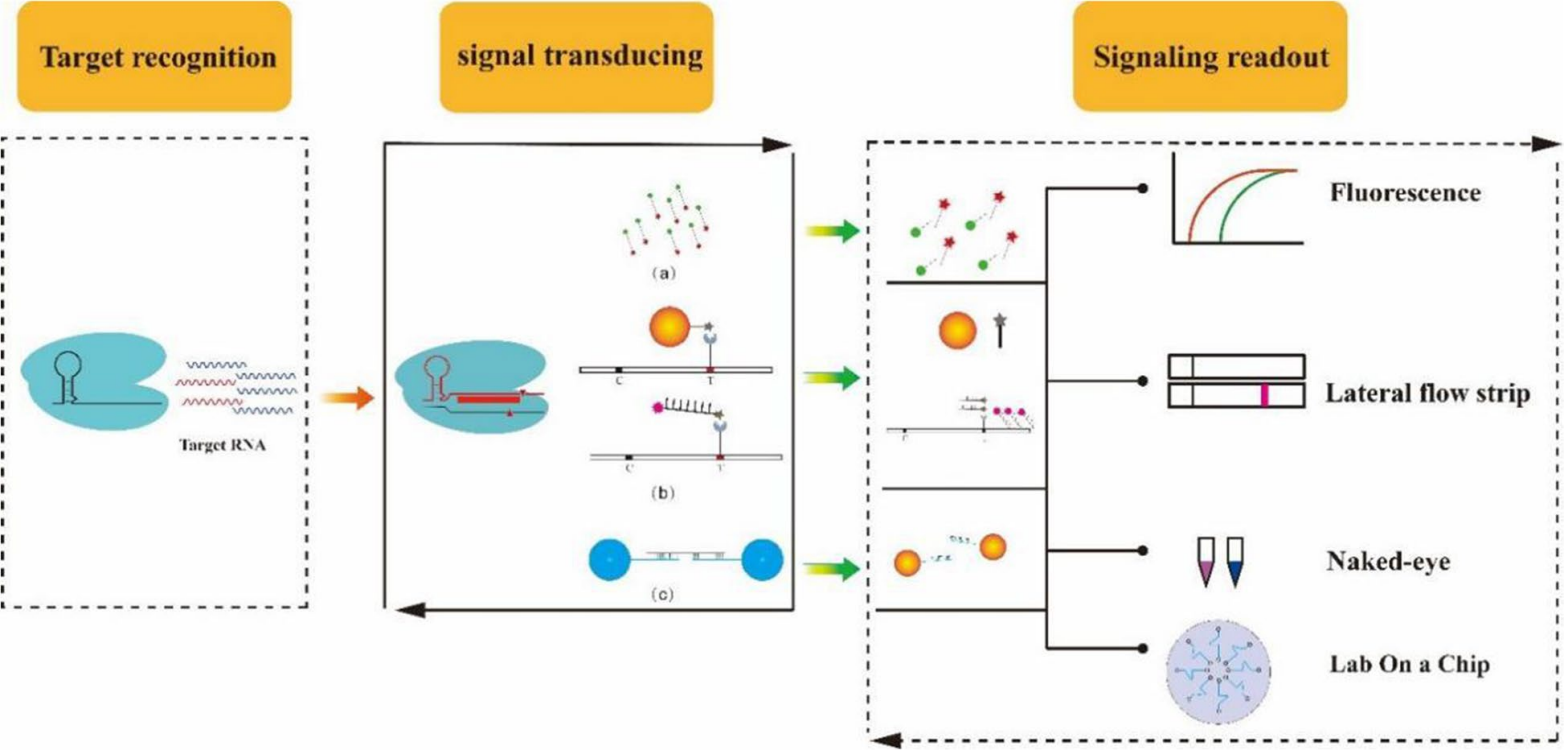
Schematics for Cas13-based Biosensing Systems

Ingenious use of the seeming shortcoming may broaden the versatility and feasibility of CRISPR/Cas13 tools, given that ultra-high sensitivity and specificity is a compelling need for many diagnostic applications, such as rapidly identified pathogen of virus disease [20, 45, 55, 56]. CRISPR/Cas13 can be used broadly to diagnose nucleic acids as lock-off switch to merely controlling signal amplification and serving to dual role that provides signal transducing and signaling amplification. Gootenberg et al. developed the first comprehensive CRISPR-based diagnostics (CRISPR-Dx) and an applicable CRISPR/Lwa/Cas13a nucleic acid detection system (Fig. 2a), termed specific high sensitivity enzymatic reporter unlocking system version 1 (SHERLOCKv1), that was immediately followed by an advanced SHERLOCKv2 system [15, 54]. In general, the comprehensive SHERLOCK system contains two feasible mechanisms: indirect detection by extracting nucleic acids for normal processing or direct detection by rapid simple pre-treatment of pathogen samples to release nucleic acids using heating unextracted diagnostic samples to obliterate nucleases (HUDSON), along with four key steps including **1)** preparing nucleic acid specimens; **2)** rapid nucleic acids amplification by RT-RPA; **3)** T7 transcription of ssRNA to amplicons; and **4)** signaling readout of Cas13a detection [15, 54]. SHERLOCKv1 has been utilized to sensitively detect ZIKV and DENV in clinical isolates where titers can be as low as 2 × 10^3^ copies/mL [46, 54]. Nevertheless, preliminary SHERLOCKv1 is monotonic and premature, lacking abundant diagnostic and commercial consideration applications. In comparison, four advances were integrated into SHERLOCKv2 by using two subgroups of Cas12a and subtypes of Cas13 to differentiate the multiplexed detection process and signaling readout. These included **1**) four-channel single-reaction multiplexing with orthogonal CRISPR enzymes; **2**) quantitative measurement of input as low as 2 aM; **3**) 3.5-fold increase in signal sensitivity by combining Cas13 with Csm6; and **4**) lateral-flow readout. Synchronously, SHERLOCKv2 can accurately detect diverse clinical isolates of ZIKV, DENV, *Staphylococcus aureus*, and *Pseudomonas aeruginosa* (Fig. 2b) [15]. Moreover, it is feasible for the direct detection of a multivirus panel to differentiate viral species and serotypes in whole blood, serum, saliva, and urine, and permits high sensitivity (1cp/μL) and favorable veracity; however, its sensitivity in different environmental samples was approximately 3 ~  100-fold less than that for PBS. Recently, Yuan et al. reported that the combination of CRISPR/Cas13 and gold colloidal gold nanoparticle (AuNP)-DNA probes enabled naked-eye colorimetric detection of the African swine fever virus (ASFV) DNA and bacterial 16S ribosomal RNA, being also capable of quantitative analysis of individual virus samples (Fig. 2c) [13]. In addition, other effective and basic platforms have also been developed that exhibit improved sensitivity and specificity, such as electrochemical and optical signal detection [20], combined with microfluidic chips [14], nucleic acid aptamers for target recognition to promote Cas13a cleavage [57], ELISA-like CLISA [21], and other platforms [55, 58] to detect tumor-related marker miRNA, detailed in Table 2.

**Table 2:** Comparison of major platforms incorporating CRISPR/Cas-based 13

System name	Effector	Recognition type	Sensitivity	Specificity	Signal amplification	Quantitative	Multiplex	Time	Readout	Infrastructure requirement	Reference
SHERLOCKv1	LwaCas13a	DNA/Virus RNA	aM	1 nt	RPA	N	N^a^	2–5 h	Fluorescence	Substantial	[54]
SHERLOCKv2	LwaCas13a, Cca-Cas13b, Psm-Cas13b	DNA/Virus RNA	zM	1 nt	RPA	Y^b^	Y	~ 1 h	Fluorescence, Naked-eye observation	Substantial/Few	[15]
m^1^A detection	LbuCas13a	m^1^A-RNA	fM	1 nt	N	Y	N	~ 10 min	Fluorescence	Substantial	[55]
On-for-all Biosensor	LwaCas13a	Tumor marker miRNA	pM	1 nt	N	Y	N	~ 4 h	Electrochemical signal	Few	[20]
POC system	LwaCas13a	Ebola RNA	20 pfu	1 nt	N	Y	N	~ 5 min	Fluorescence	Substantial	[14]
Colorimetric platform	LwaCas13a	Virus RNA	200 copies	1 nt	N	Y	N	~ 1 h	Naked eye observation	Few	[13]

a**:** zM, 10^−21^ M or zeptomole/L; aM, 10^−18^ M or attomole/L; fM, 10^−15^ M or femtomole/L; N, no; NA, not applicable; nt, nucleotide; Y, yes. b: Scale level quantitative results achieved. CLISA**:** CRISPR/Cas13a signal amplification-linked immunosorbent assay

### Cas12 Biosensing Properties and Applications

#### Collateral Sensors Based on CRISPR/Cas12

Recently, by triggering crRNA-complementarity with dsDNA substrates, Class 2 proteins of Type V-A Cas12 (homologues of AsCas12a, FnCas12a, LbCas12a, respectively) have been utilized to potential diagnostic effectors as novel biosensor. Furthermore, Cas12a orthologs of AsaCas12b (*Acidaminococcus* sp.*)* provide extended similar utility [33, 34]. Those currently reported CRISPR/Cas12 biosensing systems can be classified into two groups based on the different Cas12 effectors and signaling readout. The typical establishments and comparison of major features for these two classes is listed in Table 3. For diagnostics process, obviously crucial points of target recognition with guide RNA (gRNA), signal transducing and amplification by nonspecifically nuclease of Cas12 carried out respectively [64]. High thermal stability still maintain normal collateral cutting activity at 55 °C that provide ability of tolerance to endure trenchant chemical and physical circumstance for biochemical processing of those genome editing and diagnostics [65]. Accuracy target recognition and unleash indiscriminate DNA cutting stems from a minimum of 15 nt crRNA complementarity with the target DNA. On the one hand, the requirement of a 5′-TTTN PAM may limit the availability of high GC content at suitable target sites, reducing the practical utility of Cas12, oppositely, target specifity can be enhanced by corresponding to prolong gRNA spacer sequence. In general, the 5′-TTTN PAM sequence required for dsDNA binding by CRISPR/Cas12 is critical for catalytic activation by a crRNA-complementary dsDNA, in contrast to a crRNA-complementary ssDNA. The genome editing activity of FnCpf1 in human cells is reported to be lower than that of Cpf1 proteins from *Acidaminococcus* sp. BV3L6 (AsCpf1) and *Lachnospiraceae* bacterium ND2006 (LbCpf1), but its off-target rate is negligible. Moreover, the PAM sequence (TTN) of FnCpf1 is shorter than that (5′-TTTN) of AsCpf1 and LbCpf1 [66]. Chen et al. also demonstrated that AaCas12b and LbCas12a afforded high specificity and cleavage activity among Cas12 homologs with regard to dsDNA distinguishability, whereas relatively poor degradation efficiency was obtained with PrCas12a when 5′-TTN was used as a component of PAM [67]. Inclusion of the Cas12-crRNA spacer at the mismatch position of 9 ~ 20 nt still maintained triggered cleavage activity, which likely led to false positive detection [68]. Thus, it is essential that when designing SNP site, focus should be placed on nt positions 1 ~ 8, which support specific and conservative activation. A reliable model of Cpf1-RNA DNA recognition binding includes formation of an R-loop, cleavage, and product release, which represents the intrinsic basis of specificity and sensitivity in detection applications. Specifically, the cleavage process strongly depends on rate-limiting binding that impedes destabilization of the R-loop complex when mismatch occurs within the nt 1 ~ 8 seed region [68]. Notably, target strand (TS) cleavage occurs more slowly than collateral cleavage of the non-target strand (NTS). Cpf1 requires fewer PAM-proximal matches for cleavage when the mismatched region is pre-unwound, with numerous reports demonstrating that non-specific cutting occurred when any single mismatch in the spacer (1 ~ 20 nt) exhibits recognition of target DNA to trigger trans-ssDNA cleavage. This indicates that the Cas12 enzyme may be unsuitable as an effector for direct ssDNA detection in commercial applications. Additionally, in contrast to a bound ssDNA activator, trans-ssDNA cleavage of dsDNA targets exhibits high activity, catalyzing approximately 1250 turnovers per second [65, 69]. Based on the unique properties of Cas12 RNA-guided endonucleases, diverse platforms have been created to detect nucleic acid biomarkers related to tumors [70], small organic molecules as markers of disease [59], and infectious pathogens such as *Mycobacterium tuberculosis* [63], ASFV [61, 71], and SARS-coronavirus-2 (CoV-2) [19], at high specificity and sensitivity.

**Table 3:** Comparison of major platforms incorporating CRISPR/Cas 12

System name	Effector	Recognition type	Sensitivity	Specificity	Signal amplification	Quantitative	Multiplex	Time	Readout	Infrastructure requirement	Reference
CaT-SMelor	LbCas12a	Small molecule	nM	Single molecule	N	Y	N	40 min	Fluorescence	Substantial	[59]
OCTOPUS	LbCas12a	dsDNA	One copy	1 nt	RPA	Y	N	50 min	Fluorescence	Substantial	[60]
CORDS	LbCas12a	ASFV	aM	1 nt	RAA	N	N	~ 1 h	LFA	Few	[61]
Cas12aVDet	LbCas12a	Mycoplasma	aM	1 nt	RPA	N	N	~ 30 min	Naked eye	Few	[25]
CDetection	AaCas12b	HPV	aM	1 nt	RPA	N	N	~ 2.5 h	Fluorescence	Substantial	[62]
CRISPR-MTB	LbCas12a	TB	98%	1 nt	RPA	N	N	/	Fluorescence	Substantial	[63]
DETECTR-COVID19	LbCas12a	SARS-CoV-2	95%	1 nt	RT-LAMP	N	N	~ 45 min	LFA	Few	[19]

CaT-SMelor: CRISPR-Cas12a- and aTF-mediated small molecule detector. OCTOPUS: One-pot toolbox with precision and ultrasensitivity; higher sensitivity than general qPCR; LFA: Lateral flow assay; ASFV: African swine fever virus; CORDS: Cas12a-based on-site and rapid detection system; Cas12aVDet: Cas12a-based visual detection; CRISPR-MTB: Clinic test; CDetection: Cas12b-mediated DNA detection; DETECTR: CRISPR trans reporter

### Applications of Cas12 Endonucleases for Nucleic Acid Detection

**Fig. 3:**
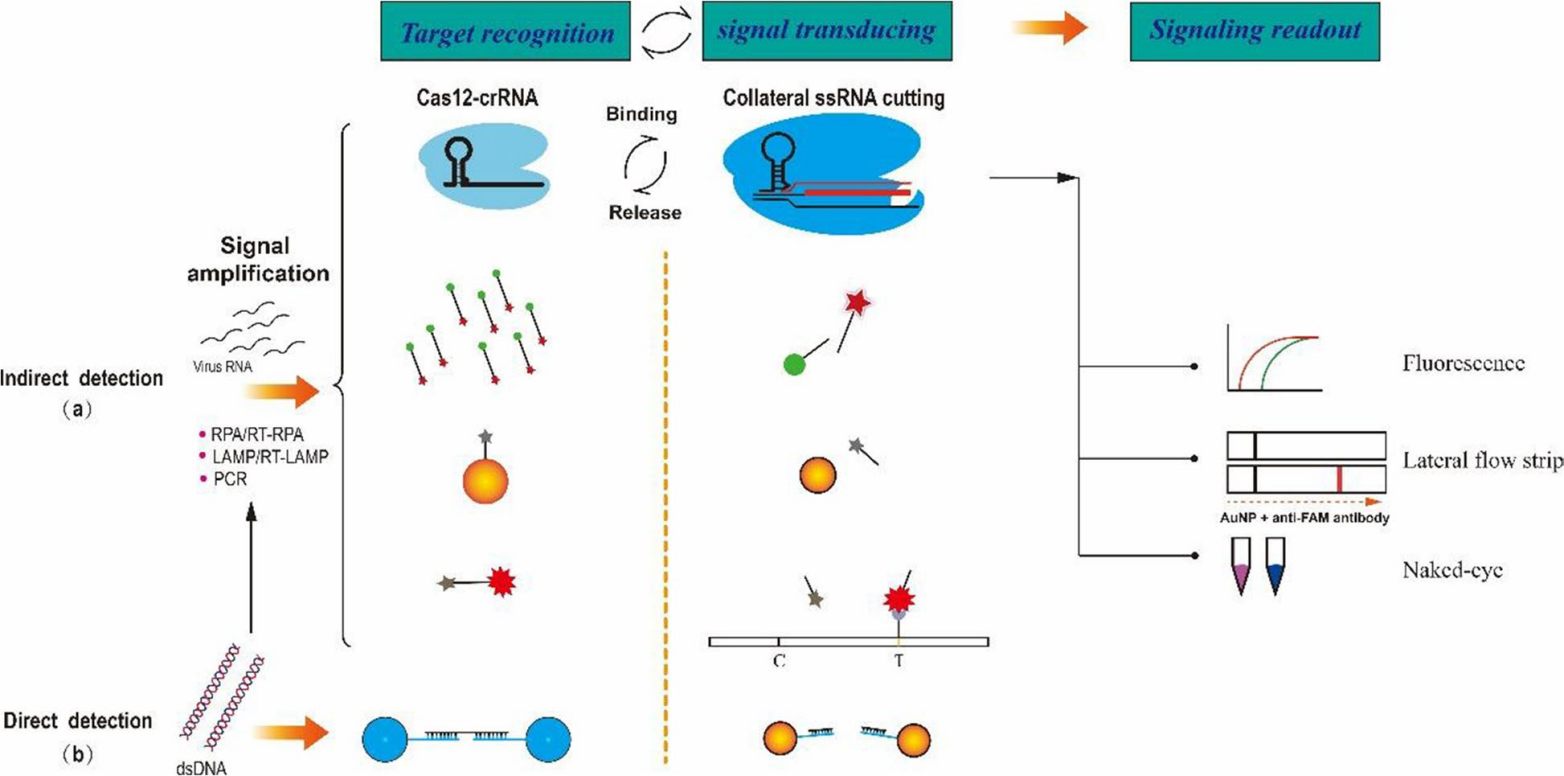
Schematics for Cas12-based Biosensing Systems

Gradually, its wide adaptability of Cas12 effector have been ensured by applied in reportedly advanced multiple of pathogens detection, indeed established profound prospects of progress [19, 25, 63, 71, 72]. Concretely, the detection process can be reached to summarized these steps, firstly an active Cas12 nuclease turns over multiple dsDNA substrates that is a biochemical behavior to be leveraged to coupling two process both signal transducing and amplification of discriminating activation to non-specific ssDNA reporter cleavage, meanwhile there is compact adjoint to explosively exponential liberation with quenched fluorophore or in an onsite manner by lateral-flow strip, finally signaling readout was captured to provide quantitative analysis (Fig. 3a). For instance, Chen et al. reported that the DNA endonuclease targeted CRISPR trans reporter (DETECTR) system for accurate and rapid detection of human papillomavirus (HPV), in which LbCas12a is used to genotype HPV types 16 (HPV16) and 18 (HPV18) that account for the majority of precancerous lesions. In particular, specific LbCas12a-crRNAs related to HPV are used as recognition tools to transduce the detection signal [24]. The ssDNA FQ reporter acts to produce a signal only in the presence of the cognate target, being degraded to achieve signal amplification to enhance sensitivity. Toward this end, isothermal amplification by recombinase polymerase amplification (RPA) was coupled with LbCas12a to identify HPV with aM sensitivity. For example, using HOLMES (a 1-h low-cost multi-purpose high-efficiency system), Wang et al. established a detection platform based on Cas12a [18]. This method uses LbCas12a, FnCas12a, and Cas12b to achieve accurate detection in 1 h and can quantify DNA/RNA viruses and provide genotyping. Moreover, it is activated similarly to DETECTR albeit combines symmetric PCR (HOLMESv1) and loop-mediated isothermal amplification (LAMP) (HOLMESv2) to enhance accuracy [73]. Furthermore, visualization tools of signaling readout using lateral-flow strips have been developed to successfully detect multiplex pathogens, e.g., containing sensors for non-nucleic-acids target of CaT-SMelor to analyze small organic molecules [59, 74], one-pot toolbox with precision and ultrasensitivity (OCTOPUS) [60], CORDS (Cas12a-based on-site and rapid detection system) for ASFV and DETECTR-COVID19 (as shown in Table 2). The use of such tools has indicated that the cleavage activity of AaCas12b is similar to that of LbCas12a, whereas both are better than PrCas12a. These data indicate that such platforms demonstrate better sensitivity and more rapid readout than those of traditional tools such as PCR or qPCR, thereby providing an alternative method to recognize and visualize nucleic acid biomarkers. Moreover, a direct, low-cost application using Cas12 is provided by the colorimetric DNA detection platforms created by Yuan et al., which represent creative technology that couples an LbCas12a effector and probes based on AuNPs-DNA (Fig. 3b) [72]. In particular, the aggregation and dispersion behaviors of AuNPs exhibit unique distance-dependent optical properties that change the color of a colloidal solution from red to purple, which can be clearly identified with the naked eye. Complete characteristics of the typical processes of the different platforms are shown in Table 2. Creatively advanced progress, C-Brick method of details was reported that Cas12a of targeting cleavage can be basically digested tools to accuracy provided cohesive end functioned as editing vector of plasmid, and then the restriction fragments were ligated with T4 ligase, thus its establishments of digested applications to plasmid that replaced possibly traditional DNA restriction endonuclease [75].

## Unique ssDNA DNase Activity and Applications of Cas14

Recent advance in use of the CRISPR/Cas enzyme for diagnostics was reported by Harrington et al., who described a novel Class 2 protein of the Type V Cas14 family distantly related to type V-U3 Cas proteins in uncultivated archaea characterized by small, exceptionally compact RNA-guided nucleases (400 ~ 700 amino acids) [26, 76]. Despite their small size, Cas14 proteins are capable of targeted ssDNA cleavage without restrictive sequence requirements. Moreover, experimental data demonstrate that Cas14a1 constitutes a ssDNA-targeting CRISPR endonuclease that may not require a PAM for activation. Notably, mismatches near the middle of the ssDNA target strongly inhibited Cas14a activity, which served to benefit the signal readout of CRISPR/Cas14 detection. Target recognition by Cas14 triggers non-specific cutting of ssDNA molecules and achieves high fidelity ssDNA detection for a HERC2 eye color single nucleotide polymorphism (SNP) using Cas14-DETECTR. In contrast, the class II CRISPR/Cas system was shown to improve molecular diagnostics afforded by Cas14a for a wide range of 100 ~ 200 nt targets, such as direct rapid detection of ctDNA, cfDNA, cffDNA, cell-free donor DNA for non-invasive prenatal testing (NIPT), and liquid biopsy. Such tests variously related DNA size in plasma with cancer, pregnancy, transplantation, and autoimmune disease. It is possible that RNA was transformed into ssDNA using first strand synthesis by reverse transcriptase.

## Comparison of Diverse Cas Effectors

To date, Cas9, Cas12 (including Cas14), and Cas13 are the only Class 2 CRISPR-Cas effectors for which ternary complex structures along with gRNA and target DNA or RNA are available to support plentiful detection applications with reasonable accuracy. In particular, diverse systems utilizing Cas9 as an effector and novel protocols including Cas13 have been established. The majority of the reported CRISPR/Cas biosensing systems afford advantages of simplicity in development/re-development, ultra-high resolution of single-base variations, sensitivity of at least fM or mostly aM concentrations, and in some cases the lack of requirement for dedicated instruments. Additionally, these nucleic acid biosensing systems have the potential to satisfy the various needs of POCT (point-of-care testing), field deployment diagnostics, in addition to those of conventional molecular diagnostics, as they can be modified to function with a variety of in vitro media efficiently and robustly. Nevertheless, off-target effects in CRISPR/Cas system remain a major concern for CRISPR-based applications, because stability and reliability are highly essential for diagnostic technology related to pathogens, especially during epidemic outbreaks. High sensitivity and specificity, however, of reported CRISPR/Cas system-based detection depend on nucleic amplification strategies such as RPA, PCR, and LAMP, whereas traditional detection technologies offer advantages with regard to cost and long-term accreditation in the research community. Although direct detection using Cas proteins as biosensors can obviously reduce detection cost and simplify the detection process, it provides low sensitivity and specificity when performed without preamplification of RPA compared to that of traditional amplification. Nevertheless, direct, low cost applications using Cas12 and Cas13 include colorimetric DNA/RNA detection platforms and fully microfluidic Ebola virus detection as developed by e.g., Yuan et al. [13], Li et al. [72], Qin team [14], respectively. Herein, similar detection process of Class 2 system is mainly composed of three steps, 1) Target recognition, reflected by pairing between the target sequence and sgRNA consisting of trans-activating crRNA (tracr-RNA), which acts to transactivate the activation complex, and crRNA with 20 nt-length recognition spacer, which serves to specifically recognize the target sequence. Cas protein recognition provides an endonuclease function that utilizes target-specific PAM or PFS (Protospacer flanking site) in the target sequence. 2) Signal transduction, by which activation of a nonspecifically degraded nuclease: R-Loop duplex with the Cas-crRNA complex target sequence triggers non-specific RNase activity. **3)** Signal amplification and readout, although generally, reported platforms based on Cas9 effectors do not involve this step. Accurate pairing between crRNA and target sequence enables degradation of the RNA reporter (e.g., RNA primer with a 5′ end-labeled reporter and 3′ end-labeled quencher), eliminating fluorescence quenching effects and releasing the 5′ end-labeled reporter. Then, the 5′ end-labeled fluorophore is excited by a laser to obtain the readout of fluorescent signal intensity. All Class 2 Cas effectors follow a similar mechanistic process and require the presence of magnesium for the cleavage reaction. The model basically comprises PAM recognition, local DNA unwinding, Cpf1-RNA–DNA recognition, binding, and formation of the R-loop, cleavage, and product release, which constitute the intrinsic bases of specificity and sensitivity in detection applications. However, although the general mechanism of the detection process is consistent across Class 2 Cas effectors, details of the cleavage patterns are distinct among Cas9, Cas12 (including Cas14 and Cas12f), and Cas13 [76]. First, the Type II CRISPR-Cas9 system accurately recognizes and cuts dsDNA but exhibits slow ssDNA cleavage activity. Conversely, Cas12 and its orthologs function as *cis*- and highly *trans*-endonucleases, providing significantly more rapid and robust ssDNA cleavage activity. Cas13 does not share similar functions with Cas12, but it is responsible only for ssRNA existence. Furthermore, PAM recognition, R-Loop formation (RNA–DNA duplex), and DNA unwinding are rate limiting for target recognition and indiscriminate cleavage, which represents a basic process for signaling amplification that directly influences its efficiency for nucleic acid detection, even under saturating conditions. CRISPR Cas effectors such as Cas9, Cas12, and Cas13, guided by sgRNA, are able to recognize and cleave any desired position but require PAM sequences adjacent to the target dsDNA or ssRNA e.g., NGG for *Streptococcus pyogenes* Cas9 (*sp*Cas9) and 5′-TTTN for *Lachnospiraceae bacterium* (LbCas12)]. Unlike Cas9 and Cas12, Cas13 recognizes the target RNA independently of the PAM sequence, instead depending on the 3′-PFS. PAM sequences are specifically recognized by Cas9, Cas12a, and Cas12b through base contacts, whereas the PFS is likely not recognized in a sequence-specific manner by Cas13. For pathogen detection, in most cases it is relatively easy to identify candidates for target sequences with suitable PAM sequences; however, fewer choices may be available when performing SNP-based discrimination and detection of other short sequences, for which the requirement of specific PAMs for each Cas effector may be difficult to satisfy. For example, the Cas9-based NASBACC method utilized a mutation in the PAM sequence to precisely distinguish the pathogen genotypes, but that may strictly limit its wider applications. Nevertheless, the kinetic framework of rate limiting binding as reflected by PAM and PFS-triggered R-Loop formation to the RNA–DNA/RNA–RNA duplex, is reversible in cases of mismatch. Moreover, Singh et al. reported that the emergence of septum post-cleavage prevents re-hybridization of DNA strands when Cas12a is locked into ultra-stable binding, whereas Cas9 exhibits weak stable binding specificity owing to only 9-bp PAM-proximal match for stable binding [65]. In contrast, Cas12a allows better fidelity through the 17-bp PAM-proximal match, potentially owing to the different abilities of the Cas enzymes to bend the DNA, creating a local kink near the PAM. Cas9 causes a larger DNA bend than Cas12a, possibly contributing to its higher tolerance of PAM-proximal mismatches with regard to binding and cleavage activity. In contrast to *sp*Cas9, Cas12a orthologs including FnCpf1, AsCpf1, and LbCpf1 exhibit slow cleavage rates. Although several mutants and strategies have been developed to improve the indiscriminate cleavage and stable binding specificity of Cas9 effector proteins, such as structurally revamped effectors of dCas9, Cas9 nickase, e*Sp*Cas9, and Cas9-HF1 along with different processes of PC reporting systems and NASBACC, the specificity remains much lower than that of Cas12a. For example, a novel diagnostic platform developed by Qiu et al. combining CRISPR-Cas9 and rolling circle amplification (RCA) to detect microRNA from non-small cell lung cancer still produced non-specific signal when detecting let-7c microRNA [22]. Detailed comparisons of multiplex channel, Cas9 in orthogonal collateral activity of dimer remains not satisfied as well understand as Cas13 **(**Table 4**)**. Nevertheless, Cas13a contains a catalytic site for RNA-guided collateral cleavage; as for Cas9 and Cas12, the active center of Cas13a is located on the external surface rather than internally. Recent advances suggest that Cas9 can be considered as a single-turnover enzyme, such that after the DNA substrate has been cleaved, the target strand remains base-paired to the guide RNA and the protein is unable to bind additional targets for subsequent reactions. Notably, this is unfavorable for efficient signal transduction when playing a continuous role in target recognition. However, Gootenberg et al. reported that LwaCas13a, CcaCas13b, and PsmCas13b are able to selectively distinguish and cleave di-AU, di-UC, and di-GA reporters, respectively, thus allowing four-channel multiplexed detection in samples of virus ssRNA and dsDNA [15]. Overall, these data indicate that Cas12 has the potential to be a better alternative to all current Cas9 variants, whereas Cas13 may serve as a better biosensor to directly detect ssRNA related to virus-associated infectious diseases or tumors, thus being better suitable for diagnostic purposes in vivo.

**Table 4:** Functional diversity of the experimentally characterized biosensing effectors of class 2 CRISPR–Cas systems

CRISPR/Cas type	Size (aa)	Nuclease domain	PAM (5′)	Stable binding specificity/bp	Substrate preference	Substrate	Cleavage pattern	Reference
Cas9	~ 1300	RuvC, HNH	NGG	9	/	dsDNA	Blunt ends	[65]
Cas12a(Cpf1)	~ 1300	RuvC, Nuc	TTTN	17	/	dsDNA	Staggered ends	[77]
Cas12b(C2c1)	~ 1100	RuvC, Nuc	TTN	17	/	dsDNA	Staggered ends	[78]
Cas13a(C2c2)	~ 1300	2 HEPN domains	Non-G, PFS	20	di-AC, di-AU	ssRNA	/	[79]
Cas13b	~ 1200	2 HEPN domains	5-A, U, G, and 3′-NAN, NNA	14	di-UC, di-GA	ssRNA	/	[52, 80]
Cas13d	~ 900	2 HEPN domains	No PFS preference	18	/	ssRNA	/	[81, 82]
Cas14a1	~ 400	RuvC, Nuc	No PFS preference	/	/	ssDNA	/	[26]

*aa* amino acid, *bp* base pair; PAM protospacer-adjacent motif; *PFS* Protospacer flanking site

## Conclusion

The expansion and improvements in CRISPR-Cas technologies have enabled more efficient and robust applications of these systems in pathogen detection and clinical diagnostic. Moreover, engineered Cas9, Cas13, Cas12, and Cas14a enzymes allow new strategies for rapid detection of tumor DNA, cancer-related viruses, and foodborne pathogens with high specificity and sensitivity and can be further optimized for detecting multiple target DNAs simultaneously. This multiplexing ability is particularly important for diagnosis, in which the genome of pathogens may exhibit a complex mutational landscape. Cas-based nucleic acid detection methods have advantages over traditional PCR and qPCR with higher sensitivity and specificity. The relatively conservative PAM sequence as the initial switch to activate the Cas protein can drive the subsequent spacer sequence to complement the target template base to form a mature R-Loop structure, specifically activating the Cas nuclease to hydrolyze the FQ reporter fluorescent molecule. The specificity derived from two unique conservative structure mechanisms is more reliable than polymerase and homosymmetric oligo nucleic acid mechanisms. Cas nuclease also has the non-specific nuclease function brought about by its specific realization process. The hydrolysis of each FQ extinction fluorescent molecule can become the basis for achieving high-efficiency signal amplification, and the process efficiency is higher. The sensitivity of traditional PCR and better qPCR depends heavily on the formation of amplified products with a size of 100 to 200 bp, increasing the detection cost. However, after combining Cas detection with RPA amplification, it can be performed at 37 °C, and detection can be achieved with only portable equipment. Further, combining this method with gold nanoparticles or dyes can realize the naked eye judgment result and reduce the dependence on optical devices. The Cas nuclease-based detection method benefits from the nuclease activity driven by the two high-efficiency PAM sequence and spacer sequence and can easily realize visual signal detection, especially in combination with the characteristics of nano-gold particles and the color of a system caused by the distance effect. The Cas system combined with PCR reduces the number of PCR amplification cycles, and overall detection time can be shortened. Before the PCR process reaches the exponential growth period, the weak signal of the amplicon produced cannot be detected. It must be accumulated to the lowest amount to be successfully detected. However, before the exponential growth period, the micro-amplification product reaches the detection amount required by the Cas nuclease detection. It replaces the relatively inefficient annealing and extension process with a more efficient non-specific nuclease activity degradation method, thereby shortening the detection time of the PCR method. Nevertheless, despite positive outcomes, these CRISPR-based detection methods still need to be tested in larger cohorts of practical specimens, optimized with regard to reagent stability, and compared side-by-side with current available technologies for pathogen diagnosis to ensure their robustness. In addition, although recently designed detection platforms based on Cas12 and Cas13 endonuclease afford improved specificity, sensitivity, and limits of detection (LOD) primarily through integrating strategies of isothermal amplification such as RPA and LAMP, they obviously require modifications in the inherent attributes of the Cas protein to enhance non-specific cleavage activity and develop novel Cas endonucleases. Additional issues remaining to be addressed include: 1) how to modify the intrinsic properties of the Cas enzyme to render a simplified enzyme size and structure that facilitates flexible packaging into size-constrained engineering strains for its extensive expression by vectors; (2) how robustly targeted genome of CRISPR-Cas systems to different sequence in complex nucleic acids of specimens will be achieved in complicated context. (3) whether side effects may result from the cleavage substrates of target DNA or RNA; (4) what efficiency might be achieved when applying these CRISPR-Cas technologies in a multiplexed manner to target and detect complex genetic alterations to accurately genotype similar types of pathogen, with minimal false-positive signals and off-target effects; and **(5)** whether universally accepted or communally normalized standards in operated processes and quality control of Cas enzymes can be defined and applied to allow these systems to serve as potential toolkits for the rapid detection of pathogens. The CRISPR/Cas detection systems can be directly used as a basic tool or combined with other technologies for nucleic acid detection, which offers the potential to significantly simplify and shorten the current nucleic acid detection process; such combinations include AuNPs, POCT, Lab-on-chip, and rapid diagnosis strips to achieve naked-eye readout. We believe that CRISPR-Cas enzyme-based systems will undoubtedly transform detection to provide a substantial clinical benefit based on the given review information.

## Abbreviations

CRISPR/Cas: Clustered regularly interspaced short palindromic repeats (CRISPR) and a sort of CRISPR-associated proteins (Cas)
dCas9: Nuclease-deactivated Cas9
PAM: Protospacer-adjacent motif sequence
PFS: Protospacer flanking site
PAMmer: PAM-presenting oligonucleotide
sgRNA: Single guide RNA
crRNA: CRISPR RNA
spCas9: Streptococcus pyogenes Cas9.
LbCas12: Lachnospiraceae bacterium Cas12
NASBACC: Nucleic acid sequence-based amplification-CRISPR cleavage.
PC: Paired dCas9.
CAS-EXPAR: CRISPR/Cas9 triggered isothermal exponential amplification reaction
RCH: RCA-CRISPR-split-HRP
RPA: Recombinase polymerase amplification
RT: Reverse transcription
HUDSON: Heating unextracted diagnostic samples to obliterate nucleases
SHERLOCK: Specific high sensitivity enzymatic reporter unlocking.
ssRNA: Single-stranded RNA.
HOLMES: One-hour low cost multipurpose highly efficient system.
DETECTR: DNA endonuclease-targeted CRISPR trans reporter.
LAMP: Loop-mediated isothermal amplification.
LFA: Lateral flow assay.
dsDNA: Double stranded DNA.
ssDNA: Single-stranded DNA.
HRP: Horseradish peroxidase.
TMB: Tetramethylbenzidine.
